# Sterolomics in biology, biochemistry, medicine

**DOI:** 10.1016/j.trac.2018.10.016

**Published:** 2019-11

**Authors:** William J. Griffiths, Yuqin Wang

**Affiliations:** Swansea University Medical School, ILS1 Building, Singleton Park, Swansea SA2 8PP, Wales, UK

**Keywords:** Mass spectrometry, Liquid chromatography – mass spectrometry, Gas chromatography – mass spectrometry, Alzheimer's disease, Niemann Pick disease, Inborn errors of metabolism, Bile acids, Oxysterols

## Abstract

In mammalian systems “sterolomics” can be regarded as the quantitative or semi-quantitative profiling of all metabolites derived from cholesterol and its cyclic precursors. The system can be further complicated by metabolites derived from ingested phytosterols or pharmaceuticals, but this is beyond the scope of this article. “Sterolomics” can be performed on either an unbiased global format, or more usually, exploiting a targeted format. Here we discuss the different mass spectrometry-based analytical techniques used in “sterolomics” giving specific examples in the context of neurodegenerative disease and for the diagnosis of inborn errors of metabolism. We pay particular attention to the profiling of cholesterol metabolites in the bile acid biosynthesis pathways, although the analytical techniques discussed are also appropriate for analysis of hormonal steroids.

## Highlights

• Sterolomics: profile all metabolites derived from cholesterol and cyclic precursors.
• Discussion of different mass spectrometry methods used in sterolomics.
• Sterolomics for disease diagnosis.
• Sterolomics and inborn errors of metabolism.

## 1 Introduction

**Fig. 1:**
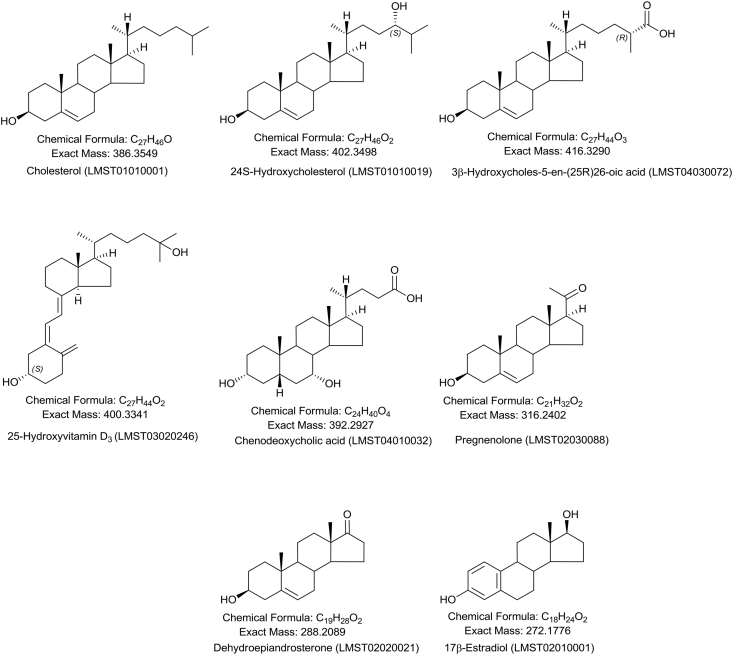
Examples of members of the sterol family. Cholesterol, 24S-hydroxycholesterol, 3β-hydroxycholest-5-en-(25R)26-oic acid, 25-hydroxyvitamin D_3_, chenodeoxycholic acid (3α,7α-dihydroxy-5β-cholan-24-oic acid), pregnenolone, dehydroepiandrosterone, 17β-estradiol.

Sterols are one of the eight classes of lipids defined by the Lipid Maps classification system [1]. This class includes cholesterol and its relatives built on the cholestane skeleton, including steroids where the 8-carbon side-chain of cholesterol is shortened, bile acids where the terminal carbon of the side-chain has a carboxyl function, oxysterols oxidised forms of cholesterol maintaining the 8-carbon side-chain and ring-opened *seco*-sterols including vitamins D_3_ (Fig. 1). The profiling of the sterol content of a system can be regarded as “sterolomics”, which is a subdivision of both “lipidomics” and “metabolomics”. Sterols are usually under-represented in un-targeted lipidomic and metabolomic studies [2] due to their poor ionisation characteristics and the dominating concentration of cholesterol and its esters. This has driven the development of “sterolomics” where sample purification schemes have been developed to specifically enrich for sterols [3], [4].

## 2 Technology

The dominating technology in “sterolomics” is mass spectrometry (MS)-based, although nuclear magnetic resonance spectrometry is unsurpassed for exact stereochemical identification of sterols. In an “omics” setting direct-infusion (DI)-MS, liquid chromatography (LC)-MS, matrix-assisted laser desorption/ionisation (MALDI)-MS, gas chromatography (GC)-MS and more recently ion-mobility-MS (IMS) and MS-imaging (MSI) all have their place for determining the global sterolome.

### 2.1 GC-MS

GC-MS has been used for decades for sterol profiling, well before the initiation of the “omics” revolution [5], [6]. It is still widely used for analysis of bile acids [7], steroids [8], cholesterol and its precursors [9] and oxysterols [10]. Many of the current methods are based on classical protocols developed in the laboratories of Sjövall [3], [6], Shackleton [11] and Björkhem [12], [13]. A pre-requisite for GC-MS analysis of sterols is derivatisation to enhance volatility and stability. Hydroxy groups are usually converted to trimethylsilyl ethers, carbonyls to methyloximes and carboxylic acid groups are methylated. Additionally, aminoacyl conjugated bile acids and steroid sulphates are enzymatically deconjugated (enzymes from *Clostridium perfringens* and *Helix promatia*, respectively) while sterols esterified with fatty acyl groups are hydrolysed or when esterified with sulphuric acid subjected to solvolysis [3], [11]. The requirements of de-conjugation and derivatisation have encouraged the movement from GC-MS to MS methods utilising desorption ionisation processes, including MALDI and the atmospheric pressure ionisation (API) method of electrospray ionisation (ESI).

### 2.2 DI-MS

DI-MS is an important technology used for screening for inborn errors of metabolism as discussed in Section 3.2. It has its origin in the classical work using fast-atom bombardment (FAB)-MS developed in the laboratories of Shackleton [11], Setchell [3] and Clayton [14] and is a precursor to “shotgun” lipidomics. In brief, samples are prepared targeting the physicochemical properties of the analytes of interest and infused into the MS, today exploiting ESI, with or without tandem mass spectrometry (MS/MS). DI-MS works most effectively for acidic or basic analytes like bile acids, steroid-sulphates or -glucuronides, or sulphates of cholesterol and oxysterols. The large changes in sterol patterns as a consequence of a defective enzyme in cholesterol biosynthesis or metabolism often makes diagnosis possible from a simple mass spectrum. Due to an absence of chromatographic separation in DI-MS mixtures of isomers are not resolved and diagnosis is based on semi-quantitative measurements or pattern recognition of MS-peaks. Definitive diagnosis is achieved by sequencing of the gene coding for the suspected defective enzyme.

### 2.3 LC-MS

LC-MS using ESI allows the analysis of polar and involatile sterols often making hydrolysis and derivatisation steps unnecessary. The ease of interfacing LC with API sources, particularly with ESI, allows the exploitation of on-line LC, making a once very challenging technology now routine. With the advent of commercial ultra-high-performance LC (UHPLC) systems, allowing back pressures of 1000 bar, and adoption of columns packed with sub-2 μm particles, high resolution chromatographic separation is now possible [15], [16]. To gain sensitivity for the LC-MS analysis of precious samples low-flow-rate-LC-MS methods are available. Capillary- (<1 mm i.d.) and nano- (<100 μm i.d.) dimension columns with flow-rates of 20–0.1 μL/min provide the benefits of concentration-dependent ESI [11], [17], [18].

### 2.4 MALDI-MS and -MSI

**Fig. 2:**
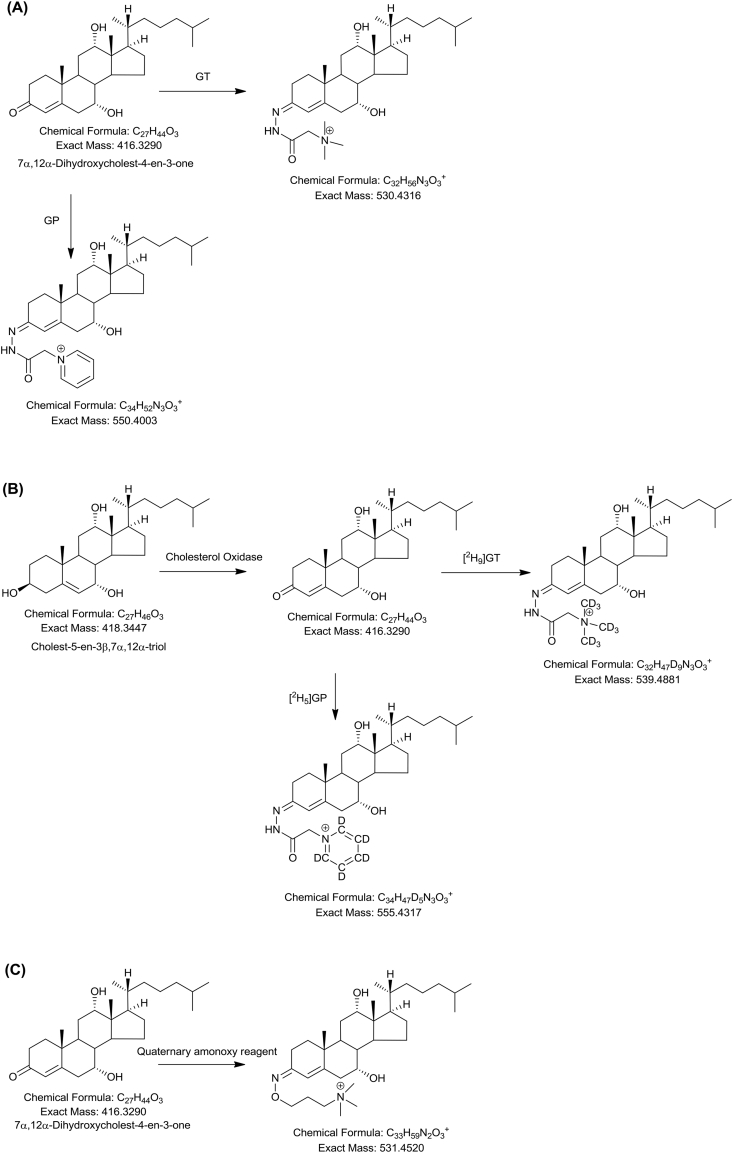
Derivatisation with charge-tagging reagents. (A) Derivatisation of a ketosterol (7α,12α-dihydroxycholest-4-en-3-one) with Girard T (GT) or Girard P (GP) reagent increases the sterol mass by 114.1026 Da or 134.0713 Da. (B) Derivatisation of a 3-hydroxy group, after oxidation to a ketone, by [^2^H_9_]GT or [^2^H_5_]GP reagent increases the sterol mass by 121.1434 Da or 137.087 Da. (C) Derivatisation of a ketosterol (7α,12α-dihydroxycholest-4-en-3-one) with (*O*-(3-trimethylammoniumpropyl) hydroxylamine increase the sterol mass by 115.123 Da.

MALDI-MS provides for another version of “shotgun” lipidomic and is being used in an increasing number of studies profiling sterols, including steroids and bile acids [19], [20]. However, it is in the realm of MALDI-MSI that the most exciting studies are being made [21], [22], [23], [24], [25], [26]. Due to their comparatively poor ionisation characteristics neutral sterols have often been derivatised to enhance their signal in MS analysis. In particular, the Girard hydrazine reagents have been exploited for analysis of sterols possessing an oxo-group, originally by FAB-MS, and latterly by ESI-MS and MALDI-MS (Fig. 2) [17], [18]. Theses reagents effectively tag a positive-charge to the sterol, hugely enhancing sensitivity. Andrew's group in Edinburgh and Ito's group in Osaka have applied the Girard T (GT) reagent to tissue material followed by matrix and proceeded to perform MALDI-MSI for GT-derivatised steroids [23], [24], [25]. Shimma et al. used this method to visualise testosterone in testis tissues of mice treated with human chorionic gonadotropin [25], while Cobice et al., also using the GT reagent, imaged steroids in mouse testis for testosterone and 5α-dihydrotestosterone at a resolution of 150–50 μm and sensitivity of 0.1 pg [24].

### 2.5 Liquid-extraction for surface analysis (LESA)-ESI-MSI

The concept of LESA for MSI derives from the work of Kertesz and Van Berkel [27] and has been cleverly exploited with on-tissue derivatisation in the area of “sterolomics” by Cobice et al. [23], [24]. The concept behind LESA is that a liquid micro-junction is created between a sampling pipette, or capillary tip, and a surface, analytes partition between the surface and the liquid which is then withdrawn and transported, either mechanically, or on-line, to an ESI emitter for MS analysis [27]. Cobice et al. used on-tissue derivatisation with the GT reagent and analysed distinct regions of mouse brain for corticosteroids [23] and androgens in mouse testis [24]. While early versions of LESA achieved resolutions of 1–2 mm, newer versions can achieve resolutions of <0.4 mm and be coupled on-line with LC.

### 2.6 Desorption electrospray ionisation (DESI) -MS and -MSI

DESI was introduced by Cooks and colleagues in Purdue in the early part of this century [28]. In brief, electrically charged droplets are electrosprayed at a target of interest under ambient conditions. Secondary ions generated from collisions of the charged droplets with the target are then sampled by the MS at atmospheric pressure. A variant of DESI is reactive DESI where a reactive reagent is incorporated in the spray solvent. Reactive-DESI has been utilised in a number of “sterolomic” investigations [29], [30]. A particularly impressive application of reactive DESI is for the MSI of cholesterol in rat brain using betaine aldehyde in the spray solvent [30].

## 3 Applications

### 3.1 Neuroscience and neurodegeneration

**Fig. 3:**
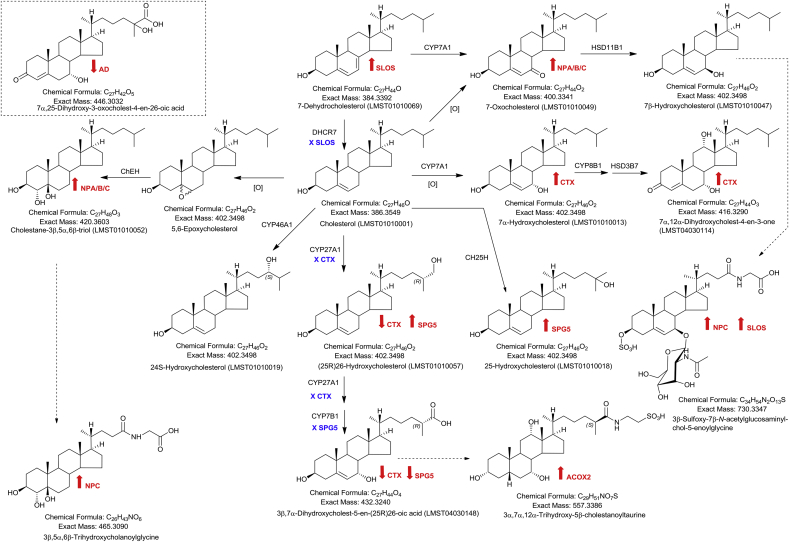
Structures of different oxysterols of potential diagnostic value. Where a defective enzyme leads to a disease, the relevant disease is indicated below the enzyme in blue preceded by an X. Where a metabolite may be diagnostic of a disease, the relevant disease is written in red, with an arrow indicating if the metabolite is of enhanced or reduced abundance. Broken lines connecting metabolites indicate that multiple enzyme catalysed reactions are involved.

About 25% of unesterified cholesterol is located in the central nervous system (CNS) of mammals [31], so it is not surprising that sterols are linked with neurological disorders. Cholesterol cannot pass the blood brain barrier (BBB), which is formed prenatally, and after birth essentially all cholesterol in the CNS is synthesised *in situ*. Excess cholesterol in the CNS is removed by metabolism, mostly to 24S-hydroxycholesterol (24S-HC, Fig. 3) which can pass the BBB. The BBB is permeable to oxysterols and cholestenoic acids which can pass into and out of the CNS [32].

#### 3.1.1 Alzheimer's disease (AD) and Parkinson's disease (PD)

Apolipoprotein E (APOE) is the principal cholesterol carrier in brain. The ε4 allele of the *APOE* gene is the most robust genetic risk factor for sporadic AD. Recently, large genome-wide association studies have classified cholesterol metabolism-related genes, including, *ABCA7* (ATP binding cassette subfamily A member 7), *ABCG1* (ATP binding cassette subfamily G member 1), *CLU* (apolipoprotein J) and *SORL1* (LDLR relative with 11 ligand-binding repeats), as susceptibility loci [33], while Picard et al. have found expression levels of the rs2269657 allele of *SREBF2*, the gene that codes for sterol regulatory element-binding protein-2 (SREBP-2), in frontal cortex from late-onset AD brain to be inversely correlated with plaque density and with age at death [34]. In combination this data strongly links cholesterol biochemistry to AD.

Oxysterol levels have been measured in cerebrospinal fluid (CSF) of AD patients and controls mostly by GC-MS methods which include a hydrolysis step to hydrolyse fatty acyl esters. 24S-HC was found to be elevated in CSF from AD patients [35]. This was explained by enhanced degeneration of neuronal cells leading to increased levels of 24S-HC in the CSF [35]. In contrast, GC-MS studies found a reduction of 24S-HC in plasma of AD patients. This apparent contradiction is explained by a reduced flux of 24S-HC across the BBB, with plasma 24S-HC concentrations reflecting the reduced number of metabolically active neurons [35]. In a recent study using LC-ESI-MS methods incorporating Girard P (GP)-derivatisation we found no difference in the level of non-esterified 24S-HC in AD and control CSF. The concentration of the non-esterified molecule in CSF is extremely low (0.02 ng/mL) making accurate measurement of small changes difficult. However, cholestenoic acids are more abundant in CSF and we have recently found 7α,25-dihydroxy-3-oxocholest-4-en-26-oic acid (7α,25-diH,3O-CA, Fig. 3) to be reduced in AD CSF compared to controls.

C_24_ bile acids have also been implicated in AD. Using LC-MS/MS methods Marksteiner et al. found levels of lithocholic acid were significantly enhanced in plasma of AD patients (50 ± 6 nM, p = 0.004) compared to healthy controls (32 ± 3 nM) [16]. This is a particularly interesting finding as lithocholic acid is usually considered as a secondary bile acid derived from bacterial reduction of chenodeoxycholic acid. In contrast, Pan et al., also using LC-MS/MS, found significantly lower cholic acid concentrations in plasma of AD patients compared to age-matched control subjects (p = 0.03), but did not find enhanced lithocholic acid levels [15]. Like-wise, the findings of Pan et al. [15] were not confirmed by the data of Marksteiner et al. [16]. More careful studies are required to remedy these contradictions. Perhaps, the modern investigator should re-consider their methods of bile acid extraction and remember the great care taken by earlier workers in this regard [3], [6].

Many studies have linked abnormal lipid metabolism and lysosomal dysfunction to PD [36]. In this context Björkhem and colleagues, again using GC-MS following hydrolysis, found that plasma levels of 24S-HC and (25R)26-hydroxycholesterol (26-HC) were in the normal range, however, in CSF the level of 24S-HC correlated with disease duration, although 24S-HC levels in 90% of patients were within the normal range of concentrations [37]. Cheng et al., also using GC-MS following hydrolysis, analysed the oxysterol content of PD brain [38]. Of the regions analysed, the visual cortex (VC) showed the most significant changes in oxysterol levels [38]. Cheng et al. found increases in 7α-hydroxycholesterol (7α-HC), 7β-hydroxycholesterol (7β-HC), 5β,6-epoxycholesterol (5β,6-EC), 7-oxocholesterol (7-OC), 26-HC and 24S-HC in PD VC (Fig. 3). While 24S-HC and 26-HC are formed enzymatically from cholesterol by the cytochrome P450 (CYP) enzymes CYP46A1 and CYP27A1, respectively, and both enzymes are expressed in brain, 7α-HC, 7β-HC and 7-OC can be formed by CYP7A1. CYP7A1 is liver specific and will 7α-hydroxylate cholesterol to give 7α-HC, introduce a 7-oxo group to 7-dehydrocholesterol (7-DHC) to give 7-OC, which can be then be reduced by hydroxysteroid dehydrogenase (HSD) 11B1 to 7β-HC [39]. These three sterols could conceivably cross the BBB and enter the brain from the circulation. Alternatively, 7α-HC, 7β-HC, 7-OC and also 5,6-EC can be formed by non-enzymatic oxidation reactions of cholesterol perhaps *in vivo* as a consequence of oxidative stress or *ex vivo* during sample handling procedures.

Gaucher's disease is an autosomal recessive lysosomal storage disorder. It is caused by mutations in the glucoceribrosidase (*GBA*) gene. Mutations in *GBA* constitute the most common risk factor identified for PD to-date [40]. Another autosomal recessive lysosomal storage disorder, Niemann-Pick (NP) type A and B, which results from mutations in the sphingomyelin phosphodiesterase 1 (*SMPD1*) gene is also reported as a risk-factor for PD [41], while heterozygous carriers of the autosomal recessive lysosomal storage disorder NP type C1 (NPC1), with mutations in the *NPC1* gene, have also been reported to present with PD [42]. Furthermore, oligomeric α-synuclein associated with PD development is increased in plasma from patients with Gaucher's disease, NPC and also Wolman disease (infant lysosomal acid lipase, LIPA, deficiency), yet another lysosomal storage disorder [43]. Cellular cholesterol is reported to influence the severity of Gaucher's disease [44], while patients with NPA, NPC, Wolman disease and carriers of NPC1 each show elevated plasma levels of oxysterols derived from non-enzymatic reactions, or their down-stream metabolites, perhaps implicating these molecules with PD [39], [45], [46]. These data point to a need for large scale studies of patients with Gaucher's disease and those with PD to investigate levels of non-enzymatically derived oxysterols and their metabolites.

#### 3.1.2 Motor neuron disease

Oxysterols have been repeatedly linked to motor neuron neurodegeneration [47]. There is convincing evidence for their involvement in the loss of motor neurons, particularly in the disorder hereditary spastic paraplegia type 5 (SPG5). SPG5 is a rare autosomal recessive disorder caused by mutations in *CYP7B1*, the gene encoding oxysterol 7α-hydroxylase, and SPG5, like other hereditary spastic paraplegias, is characterised by progressive neurodegeneration of corticospinal tract motor neurons. SPG5 is biochemically defined by elevated levels of 25-hydroxycholesterol (25-HC), 26-HC and 3β-hydroxycholest-5-en-(25R)26-oic acid (3β-HCA) in serum/plasma and CSF [47], [48], [49]. In a recent study, using GC-MS following base hydrolysis, Schöls et al. found that the serum concentration of 26-HC, measured by GC-MS as the sum of its esterified and non-esterified forms, correlated with disease severity and duration [47]. Furthermore, 24S-HC, 25-HC, 26-HC and 3β-HCA were found to be cytotoxic towards cortical neurons derived from human induced pluripotent stem cells (iPSCs) and a motor neuron-like cell line NSC-34 [47]. *In vivo* and *in vitro* studies by Theofilopoulos et al. have shown that 3β-HCA is toxic towards oculomotor neurons in mouse, while the product of its metabolism by CYP7B1, 3β,7α-dihydroxycholest-5-en-(25R)26-oic acid (3β,7α-diHCA), is protective towards oculomotor neurons in the developing mouse [48]. Interestingly, the 7β-epimer, 3β,7β-dihydroxycholest-5-en-(25R)26-oic acid (3β,7β-diHCA), which is not down regulated in SPG5 human plasma or CSF, was toxic towards oculomotor neurons in mice [48]. 3β,7β-diHCA is likely to be formed from 3β-hydroxy-7-oxocholest-5-en-(25R)26-oic acid (3βH,7O-CA) in a reaction catalysed by the enzyme HSD11B1 [39]. 3βH,7O-CA was found to promote the maturation of oculomotor neurons in developing mouse and zebrafish [48]. In a recent “sterolomic” study performed by Abdel-Khalik et al. using LC-MS with multistage fragmentation (MS^n^) and exploiting GP-derivatisation technology, the concentration of non-esterified cholesterol was found to elevated in CSF of patients suffering from amyotrophic lateral sclerosis (ALS), the most common form of motor neuron disease, and when normalised to cholesterol the concentrations of 3β,7α-diHCA and 3β,7β-diHCA were found to be reduced, so was 3β-HCA but not 26-HC [50]. Both the studies performed by Theofilopoulos et al. and Abdel-Khalik et al. exploited LC-MS(MS^n^) with GP-derivatisation [48], [50].

### 3.2 Inborn errors of metabolism

In Section 3.1.1 the autosomal recessive inborn errors of metabolism classified as NP disease were referred to. NPA (presenting in early infancy) and NPB (presenting in children) are both caused by mutations in the *SMPD1* gene, while NPC1 and NPC2 are caused by mutations in the genes *NPC1* and *NPC2*, respectively. The NP diseases are characterised by enhanced levels of oxysterols derived by non-enzymatic oxidation reactions. Analysis of plasma for the primary metabolites, 7-OC and cholestane-3β,5α,6β (3β,5α,6β-triol), by either GC-MS or LC-MS/MS with derivatisation, can diagnose NP diseases [51], [52], [53]. However, both metabolites can be generated *ex vivo* from reaction with atmospheric oxygen, so neither is ideal for disease diagnoses. 7-OC can be metabolised *in vivo* to 3β,7β-dihydroxychol-5-en-24-oic acid and subsequently conjugated with sulphuric acid at C-3, *N-*acetylglucosamine at C-7β and also glycine or taurine at C-24 and could provide a provide a urinary diagnostic (Fig. 3) [39], [54]. This unusual bile acid is also found elevated in urine and plasma from Smith-Lemli-Opitz syndrome (SLOS) patients (see below), however, Clayton and colleagues found that the *UGT3A1* gene, coding 7β-hydroxy-bile acid UDP *N*-acetylglucosaminyl transferase, shows a common mutation, c.T361G/p.C121G, resulting in inactivity of the enzyme [54], indicating that this is not an optimal diagnostic for NP or SLOS diseases. Alternatively, the end product of 3β,5α,6β-triol metabolism may provide a better diagnostic for NPC and Clayton's group in London and Ory's group in Washington using LC-MS/MS methods found 3β,5α,6β-trihydroxycholanoyl-glycine to be elevated in NPC plasma and dried blood spots [46], [54]. We have found the unconjugated acid 3β,5α,6β-trihydroxycholan-24-oic acid to be elevated also in patients with the autosomal recessive lysosomal storage disorder, lysosomal acid lipase deficiency (LALD), caused by mutations in the *LIPA* (lysosomal acid lipase) gene [45].

Two other autosomal recessive inborn errors of metabolism, presenting with quite different clinical features from the lysosomal storage disorders, but also showing elevated plasma levels of 7-OC in some patients are SLOS and cerebrotendinous xanthomatosis (CTX) [55]. In the case of SLOS, where the cause of the disorder is deficiency in the enzyme 7-dehydrocholesterol reductase (DHCR7) and main biochemical feature is elevated levels of the cholesterol precursor 7-DHC, the origin of 7-OC is likely to be the enzymatic oxidation of 7-DHC to 7-OC by CYP7A1 [39]. In SLOS, like NPC, 7-OC can be metabolised to 3β,7β-dihydroxycholest-5-en-24-oic acid and its conjugates. SLOS is usually diagnosed by an elevated 7-DHC to cholesterol ratio in plasma, either by GC-MS or LC-MS/MS, but conjugated 3β,7β-dihydroxycholest-5-en-24-oic acid may provide a urinary diagnostic, perhaps even from the mother prenatally. In CTX, caused by deficiency in the enzyme CYP27A1, essential to the acidic pathway of bile acid biosynthesis, CYP7A1 is up-regulated as a consequence of reduced negative-feedback by primary bile acids and it is probable that 7-OC is formed enzymatically by up-regulated CYP7A1 using 7-DHC as a substrate. CTX is usually diagnosed in adulthood, biochemically by elevated plasma levels of cholestanol using GC-MS, however, as a treatment exists (oral chenodeoxycholic acid therapy), there is a need for early diagnosis preferably from dried blood spots from infants.

deBarber and Vaz and colleagues have developed a 2 min LC-MS/MS method targeting 7α,12α-dihydroxycholest-4-en-3-one (7α,12α-diHCO) in newborn dried blood spots with only a 2 min extraction time [56]. 7α,12α-diHCO is elevated in CTX as a consequence of upregulated CYP7A1 and also sterol 12α-hydroxylase (CYP8B1). They exploited derivatisation of the 3-oxo group with (*O*-(3-trimethylammoniumpropyl) hydroxylamine) bromide to give a charged oxime (Fig. 2) [56]. The derivative gives an intense [M]^+^ ion and a strong transition for multiple reaction monitoring. An alternative derivative exploited by deBarber et al. in an earlier study is the GP reagent [57]. Most recently, Vaz, deBarber and colleagues have used MS/MS with flow-injection to diagnose CTX in a 2 min run from dried blood spots [58]. They measured the ratio between cholestanetetrol-glucuronide (probably, 5β-cholestane-3α,7α,12α,25-tetrol-glucuronide) and taurochenodeoxycholic acid. In term and pre-term infants the ratio was in the range 0–0.061 while in CTX patients the range was 0.8–866, the absence of an overlap in the ranges suggests this may provide a good diagnostic test for CTX. Zellweger syndrome is a peroxisomal disorder and can also lead to elevated plasma levels of cholestanetetrol-glucuronide but is readily differentiated from CTX by measuring the cholestanetetrol-glucuronide to taurochenodeoxycholic acid ratio, giving values similar to term and preterm infants [58].

Acyl CoA Oxidase 2 (ACOX2) is a peroxisomal enzyme involved in the side-chain shortening of C_27_ bile acid precursors to primary C_24_ acids. Vilharino et al. described the first case of human deficiency only recently [59]. The patient presented with liver fibrosis, mild ataxia and cognitive impairment [59]. The disease was diagnosed by exome sequencing. The biochemical phenotype was established by LC-MS/MS, where elevated plasma and urine levels of 3α,7α-dihydroxy-5β-cholestan-26-oic and 3α,7α,12α-trihydroxy-5β-cholestan-26-oic acids, mostly as taurine conjugates, were evident. The patient's plasma level of the taurine conjugate of the latter acid was about 25 times greater than the normal upper limit. His heterozygote parents, showed normal levels of C_27_ acids [59]. Since the first report by Vilharino et al. [59], two further patients have been diagnosed with ACOX2 deficiency [60], [61]. An adolescent boy (16 years) and his younger sister (13 years) were found by LC-MS/MS to have elevated levels of taurodihydroxycholestanoic and taurotrihydroxycholestanoic acids in serum [60]. Both the 25R- and 25S-acids were observed, suggesting a defect in ACOX2. Genetic analysis revealed a missense mutation in ACOX2 in both siblings. Surprisingly, while the male showed elevation of serum transaminases levels, a biochemical sign of liver damage, his sister exhibited normal serum transaminases levels, a fact that might reflect sex-related sensitivity to toxic C_27_ bile acids [60]. A further patient with ACOX2 deficiency has been identified who passed away at 5 months of age [61]. Using LC-MS she was found to have high plasma levels of primary bile acids consistent with cholestatic liver disease, and also elevated taurine conjugated trihydroxycholestanoic, trihydroxycholestenoic acid and tetrahydroxycholestanoic acids, suggestive of a peroxisomal disorder [61]. Genetic testing confirmed ACOX2 deficiency.

Other disorders of cholesterol metabolism and bile acid biosynthesis can be defined by LC-MS, LC-MS/MS or DI-MS, although final diagnosis should be confirmed by gene sequencing and the interested reader is directed to the excellent review by Vaz and Ferdinanusse [62].

The diagnosis of many rare inborn errors of metabolism is still hindered by the lack of available standards with semi-quantitative approaches still used for diagnosis. The field of “Sterolomics” has enabled the identification of many promising markers of disease, with an ultimate goal that these markers should move on to be used in high-quality quantitative clinical diagnostic tests.

## 4 Conclusions

“Sterolomics” is a growing field as ever more biochemical activities of cholesterol metabolites are being uncovered. The extreme diversity of cholesterol metabolism and the huge number of potential biosynthetic products dictates a need for unbiased investigations alongside more established targeted protocols. An important consideration for all investigators in the field is the degree of chemical identity reported in publications. Mass spectrometry identifications are seldom unequivocal and often rely on a knowledge of underlying biochemistry. It is recommended that all assumption are indicated in manuscripts, perhaps as part of supplemental information.

## Conflict of interest

Swansea Innovations Ltd, a wholly owned subsidiary of Swansea University has licenced the derivatization technology described in references 32, 45 and 50 to Avanti Polar Lipids Inc and Cayman Chemical.
